# Outcomes of surgical repair of Retinoschisis-associated retinal detachment compared to Rhegmatogenous retinal detachment

**DOI:** 10.1186/s12886-021-02232-7

**Published:** 2022-01-04

**Authors:** Jérôme Garneau, Mélanie Hébert, Eunice You, Alexandre Lachance, Serge Bourgault, Mathieu Caissie, Éric Tourville, Ali Dirani

**Affiliations:** 1 Faculty of Medicine, Université Laval, Quebec, Canada; 2 Department of Ophthalmology, Centre Universitaire d’Ophtalmologie, CHU de Québec - Université Laval (Hôpital du Saint-Sacrement), 1050 chemin Ste Foy, G1S4L8, Québec, Canada

**Keywords:** Outer later breaks, Pars plana vitrectomy, Retinal detachment, Retinal detachment repair, Retinoschisis, Scleral buckle

## Abstract

**Background:**

The aim of this study is to compare outcomes of primary retinal detachment (RD) repair in retinoschisis-associated RD (RSRD) and rhegmatogenous RD (RRD).

**Methods:**

This is a retrospective observational cohort study. Charts of 2247 consecutive patients operated for RD repair at the Centre hospitalier universitaire de Québec – Université Laval between 2014 and 2018 were reviewed. Patients with RSRD and RRD were included to compare the visual and anatomical outcomes of both groups.

**Results:**

There were 41 patients (1.8%) with RSRD and 1661 patients (74%) with RRD. RSRD patients had more primary repair failures (*n* = 9, 22%, vs. *n* = 166, 10%; *p* = 0.013). The primary anatomical success rates for pars plana vitrectomy with and without scleral buckle (PPV-SB vs. PPV) as primary repair method were similar in both RSRD patients (*n* = 11/14, 79% vs. *n* = 20/25, 80%; *p* = 0.92) and RRD patients (*n* = 751/827, 91% vs. *n* = 641/721, 89%; *p* = 0.21). At final follow-up, best corrected visual acuity (VA) in logarithm of the minimum angle of resolution (logMAR) was 0.30 [0.10, 0.88] and 0.18 [0.10, 0.40] (*p* = 0.03) in RSRD patients and RRD patients, respectively. Presence of retinoschisis was associated with worse final VA (β 0.082, *p* < 0.001). Other predictive variables included female sex, macula-off presentation, number of RD quadrants involved, longer symptoms duration, worse baseline VA, and primary repair failure. The greatest predictors were worse baseline VA, primary repair failure, and macula-off status at presentation. Presence of retinoschisis did not significantly increase risk of primary repair failure in multivariable analysis (OR 1.45, 95% CI: 0.50–4.17; *p* = 0.49). Symptoms duration was the greatest effect factor associated with for primary repair failure (OR 1.37, 95% CI: 1.12–1.69; *p* = 0.003).

**Conclusions:**

RSRD is associated with more primary repair failure in univariate analysis, but not in multivariate analysis after adjusting for symptoms duration. It is however associated with worse final VA even after adjusting for primary repair failure. Both PPV and PPV-SB are valid repair methods for RSRD. However, RSRD remains a challenge to treat.

## Background

Degenerative retinoschisis (RS) is a disorder of the peripheral retina affecting 7% of all individuals 40 years of age and older [1]. It is characterized by a splitting between the inner and outer retinal layers and the emergence of a cystic elevation in the retina [2]. It usually remains asymptomatic but may become symptomatic in cases of extensive schisis, involvement of the macula, or in patients with outer layer break (OLB) that slowly progress to a full-thickness retinal detachment (RD). Progressive symptomatic RD arises in about 0.5% of patients with RS [3].

There are two major mechanisms through which RD occurs in RS. One mechanism occurs when both an inner layer break (ILB) and an OLB are present, allowing vitreous to penetrate the schisis cavity and pass through the breaks in the outer leaf, separating the retina from the retinal pigment epithelium. The second mechanism occurs when only an OLB is present. Fluid vitreous does not gain access to the cyst or subretinal space, therefore the detachment remains localized [4].

It is estimated that degenerative RS is responsible for 1.6% of all RD [5]. Although retinoschisis is generally asymptomatic and stable, surgical treatment is indicated in patients with progressive and symptomatic RD associated with RS [6]. Different surgery types exist for the repair of this condition, including scleral buckle (SB), pars plana vitrectomy (PPV), or pars plana vitrectomy with scleral buckle (PPV-SB). Pars plana vitrectomy (PPV) remains the most frequently used, especially for cases of OLB located posterior to the equator [7].

Few large cohorts have been reported, but the success rate of primary surgery for RD is thought to be lower in patients with RS compared to patients with rhegmatogenous retinal detachment (RRD) [7]. The aim of this study is to analyze the functional and anatomical outcomes in RS-associated-RD (RSRD) and RRD, including rate of primary surgery success. The secondary objective is to identify any factors predictive of a successful surgical outcome and better visual outcome.

## Methods

**Fig. 1:**
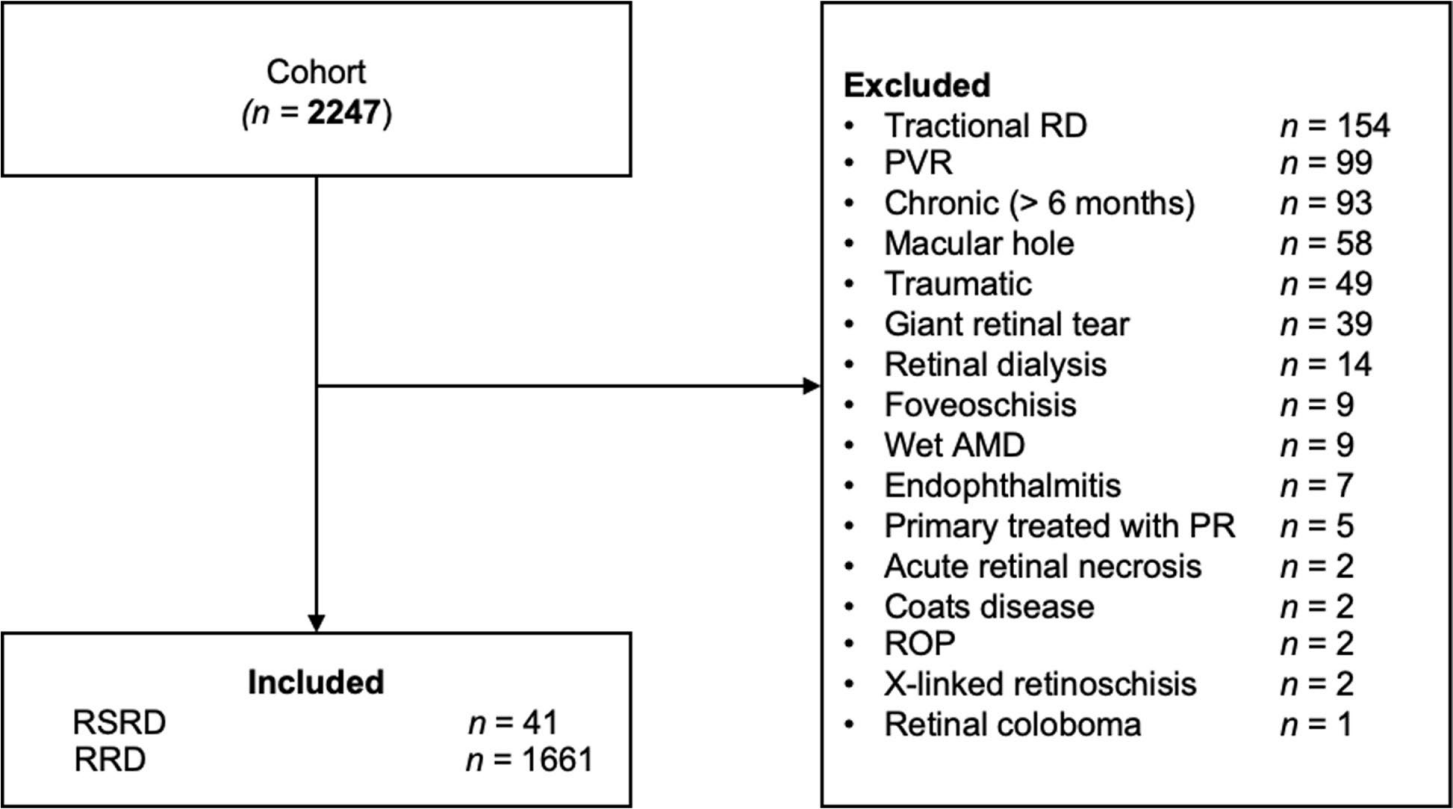
Patient selection flow chart in the cohort of retinal detachment (RD) patients. Legend. AMD: age-related macular degeneration, PR: pneumatic retinopexy, PVR: proliferative vitreoretinopathy, ROP: retinopathy of prematurity

This is a retrospective cohort study of patients with diagnoses of RSRD or RRD and adheres to the tenets of the Declaration of Helsinki. The Institutional Review Board of the Centre hospitalier universitaire de Québec – Université Laval waived the need to review this study (reference number: 2020–4798), and individual patient consent was waived. All consecutive patients operated for RD by five retinal surgeons between 2014 and 2018 at the Centre hospitalier universitaire de Québec – Université Laval (*n* = 2247) were reviewed. All 5 retinal surgeons are senior attendings with fellowship certified vitreoretinal surgery training. The distribution of the type of surgeries was comparable between all surgeons. Patients who had other causes of retinal detachment, including traumatic RD, tractional diabetic RD, RD with proliferative vitreoretinopathy (PVR grade C2 or more), RD associated with giant tears, retinal dialysis, and RD associated with macular holes were excluded. Patients treated with pneumatic retinopexy as primary surgery were also excluded (Fig. 1). We identified all patients with RSRD (*n* = 41) and RRD (*n* = 1661) in the final cohort. All ophthalmology follow-ups until March 2020 were reviewed and included. Patients had a minimal follow-up of 3 months. In our cohort, the choice of the type of surgery and the tamponade agent was at the discretion of the operating retina specialist.

We collected complete preoperative, intraoperative, and postoperative data for all included patients from baseline evaluations including visual acuity measurement, ophthalmic history, complete slit lamp exam, and dilated fundus examination, surgeon observations intraoperatively, and ophthalmologic assessments at all follow-up visits. Preoperative data included age, sex, laterality, best-corrected visual acuity at baseline, lens state (i.e. phakic, pseudophakic, or aphakic), macula state (i.e. on, off, or split), myopia greater than 4 diopters, the number of retinal quadrants affected, and the duration of symptoms before first surgery. Macula status was determined through clinical examination, but in cases which were unclear, optical coherence tomography (OCT) could be reviewed to confirm macula status. Macula on was defined as a retinal detachment not involving the macula. Retinal detachments fully involving the macula were considered macula off. A macula split occurs when a retinal detachment partially involves the macula.

The presence of RS or an RD was evaluated based on clinical assessment by a retinal specialist. Classification of patients as having RSRD or RRD, as well as classification of breaks in RS are based on the preoperative (clinical examinations) and intraoperative assessment of the RD. We defined all full-thickness retinal defects in this study as retinal tears. The number and location of retinal tears were confirmed by direct observation during surgeries. For patients with RSRD, the number of OLB and ILB was recorded. For patients with RRD, the number of retinal tears was recorded. Operative data included the type of surgery and tamponade agent used for the primary surgery. Postoperative data included information at 3 months and at the most recent follow-up such as the best-corrected visual acuity (BCVA), the development of a cataract, the retinal status (i.e. attached or detached), and the duration of total follow-up. Final BCVA were taken at patients’ last follow-up visit.

Surgical data was further analyzed to determine the influence of initial surgical choice on the primary repair failure rate. We focused on the two main surgeries used in the treatment of RD: pars plana vitrectomy (PPV) alone and PPV associated with a scleral buckle (SB) (PPV-SB) and compared their success rates. In addition, we compared the primary surgery success rate for patients with RSRD against patients with RRD. In this study, the primary surgery was considered a success if the retina remained attached at all follow-up evaluations without the need for additional surgical interventions and with a minimum follow-up of 90 days. This does not include additional laser done in the ambulatory clinic. A primary surgery was considered a failure if a patient needed a second surgery due to recurrent retinal detachment.

### Statistical analysis

Data are presented as mean ± standard deviation (SD) for continuous, normally distributed variables, as median [first quartile, third quartile] for continuous, non-normally distributed variables, and as frequencies (percentages) for categorical variables. Characteristics and variables were compared between the two groups (i.e. RSRD and RRD) using independent Student’s t-test or Mann-Whitney U test for continuous variables and using chi-square analysis or Fisher’s exact test for categorical variables, as appropriate. Shapiro-Wilk test and Q-Q plots with 95% confidence intervals were used to test for normal distribution of continuous variables. To verify whether length of follow-up had an incidence on rates of recurrence, we also compared rates of recurrence among patients who had a length of follow-up of at least 1 year.

A multivariable linear regression model was built for final visual acuity in logMAR. All preoperative characteristics were considered for inclusion in the model and all variables that could have an incidence on final visual acuity were added. To ensure approximately normal distribution of the residual errors in the model, the dependent variable of visual acuity was transformed using a square root, therefore the magnitude of regression coefficients is not directly interpretable.

A multivariable logistic regression model was built for primary repair failure. All preoperative characteristics were considered for inclusion in the model and variables that had an impact on rate of primary repair failure at the *p* < 0.20 significance level in univariable analysis were included in the final model.

Statistical analyses were performed using IBM SPSS Statistics for Windows (version 25.0; IBM Corp., Armonk, NY). All analyses were conducted at the 0.05 significance level.

## Results

### Patient baseline characteristics

During the study period, 2247 patients were operated at our center for RD. Forty-one patients (*n* = 41 eyes, 1.8%) with RD associated with degenerative RS and 1661 patients (*n* = 1661 eyes, 74%) with RRD met our inclusion criteria. Baseline characteristics by etiology are presented in Table 1. Median age at the time of surgery was significantly greater among RSRD patients compared to RRD patients (70.0 [64.5, 73.5] years vs. 62 [56, 69] years; *p* < 0.001). Nineteen men (46%) were included in the RSRD group and 1049 men (63%) were included in the RRD group (*p* = 0.03).

**Table 1:** Preoperative baseline characteristics, initial operative management, and visual outcomes at follow-up of 41 patients with retinoschisis-associated retinal detachments (RSRD) and 1661 patients with rhegmatogeneous retinal detachments (RRD)

Characteristic, n (%) or median [first quartile, third quartile]	RSRD *n* = 41	RRD *n* = 1661	***P***-value
**Preoperative characteristics**			
Age, *years*	70.0 [64.5, 73.5]	62.0 [56.0, 69.0]	**< 0.001**
Male sex	19 (46%)	1049 (63%)	**0.03**
Duration of symptoms, *days*	15 [9, 60]	7 [4, 15]	**< 0.001**
Asymptomatic patients	0 (0%)	21 (1%)	0.51
Baseline visual acuity, *logMAR*	0.30 [0.00, 0.88]	0.30 [0.10, 2.00]	0.13
*Snellen*	20/40	20/40	
Affected eye (left)	21 (51%)	805 (49%)	0.73
Myopia, > 4 diopters	3/23 (13%)	352/1620 (22%)	0.32
Lens status			
Aphakic	0 (0%)	11 (0.7%)	0.60
Phakic	31 (76%)	907 (55%)	**0.008**
Pseudophakic	10 (24%)	743 (45%)	**0.01**
Macula status			
On	25 (61%)	595 (36%)	**< 0.001**
Off	15 (37%)	884 (53%)	**0.035**
Split	1 (2%)	182 (11%)	0.08
Number of quadrants affected	2.0 [2.0, 2.0]	2.0 [2.0, 3.0]	**< 0.001**
**Intraoperative characteristics**			
Primary surgery type			
SB, n (%)	2 (5%)	113 (7%)	0.63
PPV, n (%)	14 (34%)	827 (50%)	**0.048**
PPV-SB, n (%)	25 (61%)	721 (43%)	**0.025**
Primary surgery tamponade agent			
None, n (%)	1 (2%)	30 (2%)	0.77
Air, n (%)	0 (0%)	9 (1%)	0.64
SF6, n (%)	23 (56%)	1311 (79%)	**< 0.001**
C_3_F_8_, n (%)	16 (39%)	291 (18%)	**< 0.001**
Silicone oil, n (%)	1 (2%)	20 (1%)	0.48
**Postoperative characteristics**			
Recurrence of retinal detachment	9 (22%)	166 (10%)	**0.013**
Visual acuity at 3 months, *logMAR*	0.40 [0.30, 0.79]	0.30 [0.10, 0.54]	**0.002**
*Snellen*	20/50	20/40	
Final visual acuity, *logMAR*	0.30 [0.10, 0.88]	0.18 [0.10, 0.40]	**0.003**
*Snellen*	20/40	20/30	
Follow-up time, *months*	13.0 [3.0, 30.5]	11.0 [3.0, 25.0]	0.31
Cataract development	19/31 (61%)	687/907 (76%)	0.13

Among RSRD and RRD patients, there were no differences in laterality (*p* = 0.73) or presence of myopia greater than 4 diopters (*p* = 0.32). However, in the RSRD group, more patients were phakic than pseudophakic compared to the RRD group (*p* = 0.008). Median baseline visual acuities in logMAR were 0.30 [0.00, 0.88] and 0.30 [0.00, 2.00] (20/40 in Snellen) in RSRD patients and RRD patients, respectively (*p* = 0.13).

Among the 41 RSRD patients, there were 1 [1, 2] OLB within the retinoschisis. While they all had at least one OLB, only 17 patients (41%) had at least one ILB. Those 17 patients had 1 [0, 2] ILB associated with their OLB. Among RRD patients, there were 2 [1, 3] retinal breaks. Between RSRD and RRD patients, there were more macula-on patients in the RSRD group compared to RRD group (*n* = 25, 61% vs. *n* = 595, 36%; *p* < 0.001) and there were less macula-off patients in RSRD patients (*n* = 15, 37% vs. *n* = 884, 53%; *p* = 0.035). The remainder had a split macula. The RD in RSRD patients was also less extensive with less quadrants (2 [2] vs. 2 [2, 3]; *p* < 0.001). The duration of symptoms before first surgery was significantly greater in the RSRD group compared with the RRD group (*p* < 0.001).

### Intraoperative characteristics

Choice of primary repair method varied by RD etiology (Table 1). Patients with RRD were more commonly treated with PPV compared to RSRD (*p* = 0.048), while patients with RSRD were more commonly treated with PPV-SB (*p* = 0.025). The most common tamponade agent used was SF_6_ in both RSRD and RRD patients. C_3_F_8_ was used more frequently in RSRD patients compared to RRD patients (39% vs. 18%; *p* < 0.001). Two patients (5%) and 113 patients (7%) were treated with scleral buckle alone as primary surgery in the RSRD and RRD group, respectively. In the former, one of the patients (50%) had a recurrence and in the latter, 10 patients (9%) had a recurrence (*p* = 0.18). One patient (2%) and 20 patients (1%) received silicone oil as first surgery tamponade agent in the RSRD and RRD group, respectively.

### Postoperative characteristics

The median total duration of follow-up was 13 [3, 30.5] months and 11 [3, 25] months in the RSRD and RRD groups respectively (*p* = 0.31). At 3 months follow-up, BCVA in logMAR was 0.40 [0.30, 0.79] (20/50 in Snellen) and 0.30 [0.10, 0.54] (20/40 in Snellen) (*p* = 0.002) in RSRD patients and RRD patients, respectively. At final follow-up, BCVA in logMAR was 0.30 [0.10, 0.88] (20/40 in Snellen) and 0.18 [0.10, 0.40] (20/30 in Snellen) (*p* = 0.003) in RSRD patients and RRD patients, respectively (Table 1). Among phakic patients, 19 patients (61%) developed a cataract in the postoperative period in the RSRD group compared to 687 patients (76%) in the RRD (*p* = 0.13).

Patients with RSRD were more likely to have a primary repair failure (*n* = 9, 22%, vs. *n* = 166, 10%; *p* = 0.013). Among patients who had at least 1 year of follow-up, similar results were found with a higher rate of primary repair failure in RSRD patients, though this lost statistical significance (*n* = 6/21, 29% vs. *n* = 115/801, 14%; *p* = 0.07). The primary anatomical success rates for PPV alone compared with combination PPV-SB as primary repair method were similar in both RSRD patients (*n* = 11/14, 79% vs. *n* = 20/25, 80%; *p* = 0.92) and RRD patients (*n* = 751/827, 91% vs. *n* = 641/721, 89%; *p* = 0.21). Final retina status was on for 41 (100%) of RSRD patients compared to 1655 (99.6%) of RRD patients (*p* = 0.70).

### Multivariable analysis for final visual acuity and primary repair failure

A multiple linear regression model using baseline and intraoperative characteristics was built for final visual acuity in logMAR (Table 2). Significant associations were found between worse visual acuity and female sex, presence of retinoschisis, aphakia, macula-off presentation, greater number of RD quadrants involved in the RD, longer duration of symptoms prior to consultation, and worse baseline VA. Among these, the two associations with the greatest effects were understandably worse baseline VA followed by macula-off status at presentation.

**Table 2:** Multivariable analysis for risk factors for worse final visual acuity in logMAR following retinal detachment (RD) and repair using a multiple linear regression model based on preoperative characteristics excluding RD recurrence and a model including RD recurrence; B = unstandardized coefficients, β = standardized coefficients, CI = confidence interval

Characteristic	Model excluding RD recurrence		Model including RD recurrence	
	B (95% CI); *P*-value	β	B (95% CI); *P*-value	β
Age, *years*	0.002 (0.000, 0.003); 0.053	0.052	0.001 (0.000, 0.003); 0.10	0.042
Male sex	−0.055 (− 0.088, − 0.022); **0.001**	− 0.078	− 0.054 (− 0.086, − 0.022); **< 0.001**	−0.077
Retinoschisis	0.253 (0.096, 0.409); **0.002**	0.078	0.265 (0.116, 0.415); **< 0.001**	0.082
Lens status				
Phakia	REF	REF	REF	REF
Pseudophakia	−0.032 (−0.068, 0.004); 0.08	−0.047	−0.019 (− 0.053, 0.015); 0.27	− 0.028
Aphakia	0.150 (−0.074, 0.375); 0.19	0.032	0.192 (−0.023, 0.407); 0.08	0.041
Myopia	0.009 (−0.032, 0.051); 0.66	0.011	0.015 (−0.025, 0.054); 0.47	0.018
Macula status				
On	REF	REF	REF	REF
Off	0.109 (0.064, 0.154); **< 0.001**	0.160	0.108 (0.065, 0.151); **< 0.001**	0.158
Split	0.038 (−0.019, 0.094); 0.19	0.034	0.044 (−0.010, 0.098); 0.11	0.040
RD quadrants	0.034 (0.014, 0.054); **< 0.001**	0.091	0.032 (0.013, 0.051); **0.001**	0.086
Symptoms duration, *months*	0.061 (0.031, 0.092); **< 0.001**	0.097	0.037 (0.007, 0.066); **0.02**	0.058
Baseline VA, *logMAR*	0.088 (0.069, 0.106); **< 0.001**	0.280	0.090 (0.072, 0.108); **< 0.001**	0.287
RD recurrence	N/A	N/A	0.327 (0.270, 0.383); **< 0.001**	0.264

Given that primary repair failure was significantly greater in the RSRD group, this variable was added in a second multiple linear regression model to verify if this accounted for the worse final visual acuity in RSRD patients (Table 2). Primary repair failure added value in prediction and had a greater effect size than even macula-off status at presentation. However, the other associations identified in the first model remained significant, notably presence of retinoschisis.

A multiple logistic regression model using baseline and intraoperative characteristics was used to determine risk factors for primary repair failure in this cohort (Table 3). Many of the same risk factors for final VA did not produce a similar increase in primary repair failure. Presence of retinoschisis did not significantly increase risk of primary repair failure after correction for other factors (OR 1.45, 95% CI: 0.50–4.17; *p* = 0.49), while symptoms duration was the most important predictive variable for primary repair failure (OR 1.37, 95% CI: 1.12–1.69; *p* = 0.003).

**Table 3:** Multivariable analysis for risk factors for primary repair failure following retinal detachment (RD) repair using a multiple logistic regression model based on preoperative and intraoperative characteristics; CI = confidence interval, OR = odds ratio

Characteristic	Univariable	Multivariable
	OR (95% CI); *P*-value	OR (95% CI); *P*-value
Age, *years*	1.02 (1.00, 1.03); **0.04**	1.01 (1.00, 1.03); 0.19
Male sex	0.88 (0.64, 1.21); 0.43	–
Retinoschisis	2.53 (1.19, 5.40); **0.02**	1.45 (0.50, 4.17); 0.49
Lens status		
Phakia	REF	–
Pseudophakia	0.93 (0.67, 1.27); 0.64	–
Aphakia	0.74 (0.10, 5.79); 0.77	–
Myopia	1.18 (0.79, 1.77); 0.41	–
Macula status		
On	REF	REF
Off	0.93 (0.73, 1.41); 0.93	0.96 (0.65, 1.43); 0.84
Split	0.59 (0.31, 1.12); 0.10	0.75 (0.38, 1.49); 0.41
RD quadrants	1.08 (0.91, 1.28); 0.37	–
Symptoms duration, *months*	1.46 (1.17, 1.73); **< 0.001**	1.37 (1.12, 1.69); **0.003**
Baseline VA, *logMAR*	1.06 (0.92, 1.23); 0.40	–

## Discussion

This retrospective cohort study has reviewed 2247 patients that underwent surgical intervention for RD and identified 41 patients (1.8%) with RS-associated RD. This prevalence is consistent with previous reports on RS which constituted 1.6% among a cohort of 1130 Scottish patients with RD [5]. The objective of this study was to then compare the outcomes of these patients to those who had RRD.

The baseline characteristics between the two groups differed significantly for age, sex, and lens status. Degenerative RS is an acquired age-related pathology, which explains the significantly greater mean age at surgery among patients in the RSRD group compared to the RRD group. Furthermore, it is well-known that prior cataract surgery is an independent risk factor for RRD. This would explain our findings that patients were significantly more likely to be pseudophakic in the RRD group compared to the RSRD patients despite the older age in the latter group. We also see an approximately equal proportion of males to females in our RSRD group given that degenerative RS affects both males and females equally. This contrasts with the higher prevalence of males in the RRD group which is compatible with rates seen in previous epidemiologic studies [8] and which is not entirely explained by traumatic causes [9].

Between the presentation of both groups, patients with RSRD had an indolent course with a longer duration of symptoms prior to presentation, less retinal quadrants affected, and more likely macula on at presentation. This is expected given that RSRD is usually an asymptomatic disease of the peripheral retina, becoming only symptomatic as the extent of RSRD becomes larger, starts to affect the macula, or progresses into a full thickness RD. Given these differences in baseline characteristics, in order to assess and compare the outcomes between patients with RRD and RSRD, multivariable regression models were built in order to control for confounding risk factors.

The visual acuity at 3 months postoperatively and the final visual acuity were poorer in the RSRD group compared to the RRD group. Given the more favorable preoperative characteristics (i.e. lesser extent of RD, more frequent macula-on presentation) in the RSRD group, this is a less expected finding. However, older age in the RSRD group could have contribute to the worse BCVA in this group. BCVA was also more susceptible to have worsened during postoperative follow-up in RSRD patients. The postoperative difference in visual acuity between both groups is likely caused in large part by the higher rate of primary repair failures in the RSRD group. The presence of a small macular schisis could have been also responsible of worse visual outcomes in the RSRD group during follow-up. However, even after multivariable adjustment for failure, presence of RS remains associated with worse final VA. Because the mechanism of retinal detachment in retinoschisis is not totally understood, this may be due to the underlying retinal disease among RSRD patients which could impede visual recuperation following RD repair and lead to visual deterioration. For risk of primary repair failure, RS was not found to have a direct effect after adjusting for other variables, particularly duration of symptoms. However, in our study, we must consider the effect of a small sample size in the retinoschisis group on the statistical analyses. Therefore, though the effect was not statistically significant, a clinical association cannot be excluded given the wide confidence interval.

There have been many different treatment approaches used by surgeons to manage RSRD including laser photocoagulation only, pneumatic retinopexy, SB surgery, and PPV. PPV is the most frequent surgery done for RD in RS patients with an OLB posterior to the equator [7]. In 2014, Gotzaridis et al. compared the success rate of SB alone with PPV alone in 30 patients with retinoschisis. There was a significant difference in the primary success rate between both groups, with the SB group being associated with better results. However, there was no difference in the final reattachment rate between both groups [9]. The association of PPV and SB has been also reported for the management of RD associated with RS. Sneed et al. successfully reattached the retina using PPV with gas injection in 12 RSRD patients with OLB posterior to the equator and SB was employed in 10 of the 12 patients in combination with PPV [3].

In our study, PPV alone and combination PPV-SB were the two main surgeries employed by surgeons for RSRD, with combination PPV-SB being most frequent. In RSRD patients, there were no significant difference in the anatomical success rate of first surgery between these two types of intervention (*p* = 0.92).

Our study shows that the anatomical success rate of primary surgery for retinal detachment was significantly lower in patients with RS than patients with RRD (78% vs. 90%). This is also consistent with other studies in the literature. In 2006, Grigoropoulos et al. had a primary anatomical success rate of 66% in 30 eyes with RSRD which is lower than patients with RRD [10]. Gotzaridis et al. had a primary success rate for RSRD patients of 62% with PPV and 76% with SB [11]. In contrast, in 2010, Avitabile et al. reported primary success rates of 92% in 37 patients with RSRD compared to 83% in 703 patients with RRD treated with SB [12]. It is the only large cohort study comparing the primary success rates of surgery between RSRD and RRD patients before our study. Our larger population, larger data and statistical analysis showing risk factors of primary repair failure distinguish our study from theirs.

The mechanism responsible of retinal detachment in degenerative RS is not clearly understood, but it is likely different from RRD, potentially explaining the difference in surgical success rates. In senile retinoschisis, vitreous traction chronically present in the peripheral retina might favor the development of a cystic cavity causing a schisis detachment and retinal detachment [13]. Lincoff et al. suggest the presence of intraretinal fibers located in the schisis cavity responsible of traction could also explain some reported cases of spontaneous attachment [14]. The primary success rate of surgery could also be worse in RSRD patients because of the surgery difficulty itself as retinal breaks and holes are more easily missed during procedure and can eventually be responsible for retinal redetachments.

### Limitations

This is a retrospective, single center study. We describe 41 patients with retinoschisis in this cohort, which was insufficient to build a multivariable model examining rates of primary repair failure in this group only. It likewise probably contributed to the fact that RS was identified as a predictive variable for worse final VA but not for primary repair failure given the low proportion of patients with RSRD who redetached following primary repair. Given the wide confidence interval for the effect of RS in the multiple logistic regression model (OR 1.45, 95% CI: 0.50–4.17; *p* = 0.49), the absence of an association cannot be excluded. We also could not study specific interventions to improve success rates in RSRD. However, to our knowledge, this remains one of the largest cohorts of RSRD cases reported in the literature being compared with RRD cases.

Data on outcomes was collected until the final follow-up. Rates of outcomes such as cataract development and recurrence of detachment could however be affected by length of follow-up which was restricted to 3 months in several patients. In particular, the proportion of cataract development in the RSRD group (61%) may be affected by this follow-up time, but when compared with the RRD group, both would be affected similarly given comparable lengths of follow-up.

In our study, we did not record data for presence or absence of PVD associated with RD whether in the RSRD or RRD group. In the literature, the presence of PVD was less frequently reported in RSRD compared to RRD patients [7]. This may have affected the rate of surgical success in our study [15]. Another limitation is the fact that an OCT was not routinely done in the workup of our patients and thus OCT related data has not been recorded. This could have affected the determination of the presence and localization of retinal breaks.

## Conclusion

In conclusion, our retrospective cohort study highlights that the presentation and outcomes of RD patients with RS follow a different course than that of RRD patients. Management of RD in the former also remains more challenging with anatomical and functional outcomes of surgery that are less favorable in RSRD when compared with RRD. When adjusting for other preoperative risk factors, RS is associated with worse final visual acuity but not with the rate of primary repair failure. The longer duration of symptoms was associated with an elevated rate of primary repair failure in the RS group on multiple regression analysis. Standard PPV and combined PPV-SB showed similar surgical outcomes in repairing the RD associated with a retinoschisis. More studies are needed to optimize the surgical outcome of this complex disease.

## Data Availability

The datasets used and/or analysed during the current study are available from the corresponding author on reasonable request.

## Abbreviations

BCVA: Best-corrected visual acuity
C3F8: Perfluoropropane
ILB: Inner layer break
logMAR: Logarithm of the minimum angle of resolution
OLB: Outer layer break
PPV: Pars plana vitrectomy
PPV-SB: Pars plana vitrectomy with scleral buckle
RD: Retinal detachment
RRD: Rhegmatogeneous retinal detachment
RS: Retinoschisis
RSRD: Retinoschisis-associated retinal detachment
SB: Scleral buckle
SD: Standard deviation
SF6: Sulfur hexafluoride
VA: Visual acuity
